# Tailoring Homogeneous Hydrogel Nanospheres by Facile Ultra-Sonication Assisted Cross-Linked Copolymerization for Rhodamine B Dye Adsorption

**DOI:** 10.3390/gels9100770

**Published:** 2023-09-22

**Authors:** Gaurav Sharma, Alberto García-Peñas, Yaksha Verma, Amit Kumar, Pooja Dhiman, Florian J. Stadler

**Affiliations:** 1 Shenzhen Key Laboratory of Polymer Science and Technology, Guangdong Research Center for Interfacial Engineering of Functional Materials, College of Materials Science and Engineering, Shenzhen University, Shenzhen 518055, China; mittuchem83@gmail.com (A.K.); fjstadler@szu.edu.cn (F.J.S.); 2 International Research Centre of Nanotechnology for Himalayan Sustainability (IRCNHS), Shoolini University of Biotechnology and Management Sciences, Solan 173229, Himachal Pradesh, India; angelyasiverma13@gmail.com (Y.V.); dhimanpooja85@gmail.com (P.D.); 3 Departamento de Ciencia e Ingeniería de Materiales e Ingeniería Química, IAAB, Universidad Carlos III de Madrid, Avda. de la Universidad, 30, 28911 Madrid, Spain

**Keywords:** hydrogel, dye adsorption, ultrasonication

## Abstract

The present paper describes the design of shape-oriented hydrogel nanospheres using a facile ultrasonication-supported crosslinked copolymerization technique. The effect of variable monomer concentration on the homogeneity of hydrogel nanospheres was investigated. The chitosan-*cl*-poly(MMA) hydrogel nanospheres were well characterized using various techniques such as FTIR, XRD, TGA, SEM, and TEM. The chitosan-*cl*-poly(MMA) hydrogel nanospheres were studied for their swelling behavior and could potentially be used as a novel adsorbent for rhodamine B dye remediation from aqueous media. The study found that utilizing chitosan-cl-poly(MMA) nanohydrogel spheres at the optimal pH 5 increased RhB dye adsorption capacity from 7.9 to 17.8 mg/g (pH 2 to 5), followed by a slight reduction. Furthermore, when nanohydrogel concentration increased, adsorption capacity dropped from 18.03 to 2.8 mg/g, but adsorption percentage climbed from 90.2% to 97.8%. At an initial dye concentration of 140 mg/L, rhodamine B adsorption achieved 204.3 mg/g in 60 min. The rhodamine B dye adsorption study includes adsorption kinetics, isotherm, and thermodynamics analyses. The interpretation of the adsorption study revealed that Langmuir isotherms fit best with a qmax value of 276.26 mg/g, which is in close approximation with the experimental value, whereas pseudo-second-order kinetics explains the adsorption process rate. The interaction of RhB dye with chitosan-*cl*-poly(MMA) hydrogel nanospheres involves multiple forces such as electrostatic interactions, hydrogen bonding, van der Waals forces, etc.

## 1. Introduction

With the expansion of diverse industries such as textiles, plastics, food, and pharmaceutics, water pollution caused by hazardous industrial waste, particularly dye wastewater, has emerged as a serious social and ecological concern [1,2]. The increasing use of various dyes in these sectors has contributed to the problem, as even trace levels of dyes in water can result in highly visible colored effluents that impede sunlight penetration and interrupt photosynthesis, upsetting aquatic ecology. Furthermore, the bulk of these dyes are hazardous or carcinogenic, posing risks to both aquatic life and human health over time [3]. Moreover, a look at the chromophore, or chemical structure, of dyes reveals the presence of more than 20 groups, including triphenylmethane, thiazine, phthalocyanine, oxazine, nitro, methine, nitroso, indigoid, azine, diphenylmethane, azo, anthraquinone, and acridine [4]. 

Rhodamine B (RhB) is a xanthene dye [5] that is utilized in a variety of sectors due to its high water solubility, brightness, and stability. It belongs to the xanthene dye family, and its derivatives include rhodamine 6G, rhodamine 123, and sulforhodamine 101 [6,7,8]. RhB has the chemical formula C_28_H_31_N_2_O_3_Cl with a molecular weight of 479 g/mol (IUPAC Name: N-[9-(ortho-carboxyphenyl)-6-(diethylamino)-3H-xanthen-3-ylidene] diethyl ammonium chloride), and it has a reddish-violet color [9]. Despite its advantageous characteristics, RhB is associated with serious health and environmental risks, including being neurotoxic, carcinogenic, and harmful to organismal development and endangering the ecosystem [9,10].

Several techniques have been established for the removal of RhB dyes from wastewater due to their negative effects on human health and the environment. Chemical oxidation, coagulation, and precipitation are some of the traditional treatment procedures used for dye wastewater [11]. Nonetheless, these procedures are associated with high prices and low efficiency. Aside from these approaches, advanced oxidation processes such as photocatalysis [12], electro-Fenton [13], and electrochemical oxidation [14] have been studied for the treatment of rhodamine-B-contaminated wastewater. Adsorption has received a lot of attention as a promising technology because of its low cost, high removal efficiency, and reusability [15,16]. Traditional adsorbents, such as metal oxides, activated carbons, clays, and polymers, on the other hand, have limitations such as low adsorption performance, a lack of selectivity, and poor regeneration. Because of their hydrophilic nature, biocompatibility, and renewability, hydrogels have emerged as a notable alternative for adsorbing pollutants from aquatic media [17,18,19].

Hydrogels, which are made up of a crosslinked polymeric network, retain a lot of water without disintegrating. Because of their porous nature, which allows for efficient solute diffusion and high absorption capacity, they are a promising adsorption material for pollution control and pollutant removal from water. Hydrogels have gained significant study attention in this field due to benefits such as adsorption–regeneration, economic feasibility, and environmental friendliness [20,21,22]. 

Morphological aspects at nano dimensions have most the important impact on physicochemical properties, particularly related to the application aspects of gels. Achieving shape- and size-controlled nanohydrogels is one of the most challenging tasks for present-day researchers in the context of macromolecules such as pectin, chitosan, guar gum, gum arabic, and cellulose because of their high molecular weight, hydrophilic or hydrophobic nature, and instability in different solvents. Thus, it is harder to obtain their desired morphology. Various synthesis methods, such as emulsion polymerization, hydrothermal, raft polymerization, microwave, and ultrasonication, have been explored to obtain the desired morphologies of nanohydrogels. The ultrasonication technique is a unique way to obtain controlled morphology in particles by controlling the reaction conditions and reactant concentrations. The purpose of this research is to fabricate polymeric hydrogel nanospheres for the adsorption of rhodamine B dye in an aqueous medium. Our investigation not only examines the effect of various parameters on the adsorption process but also provides significant findings, such as the pH-dependent adsorption trend, the effect of nanohydrogel concentrations on adsorption behavior, the time-dependent adsorption capacity, and the significant rise in equilibrium adsorption capacity at varying initial dye concentrations. These novel findings contribute to our understanding of the adsorption mechanism and have interesting implications for the field of dye removal utilizing advanced nanomaterials.

## 2. Results and Discussion

**Figure 1:**
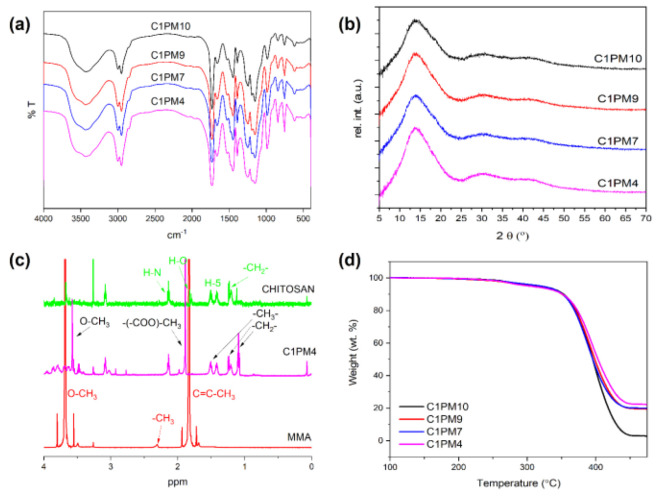
(**a**) FTIR spectra, (**b**) XRD, (**c**) NMR, and (**d**) TGA of chitosan-*cl*-poly(MMA) hydrogel nanospheres.

**Scheme 1:**
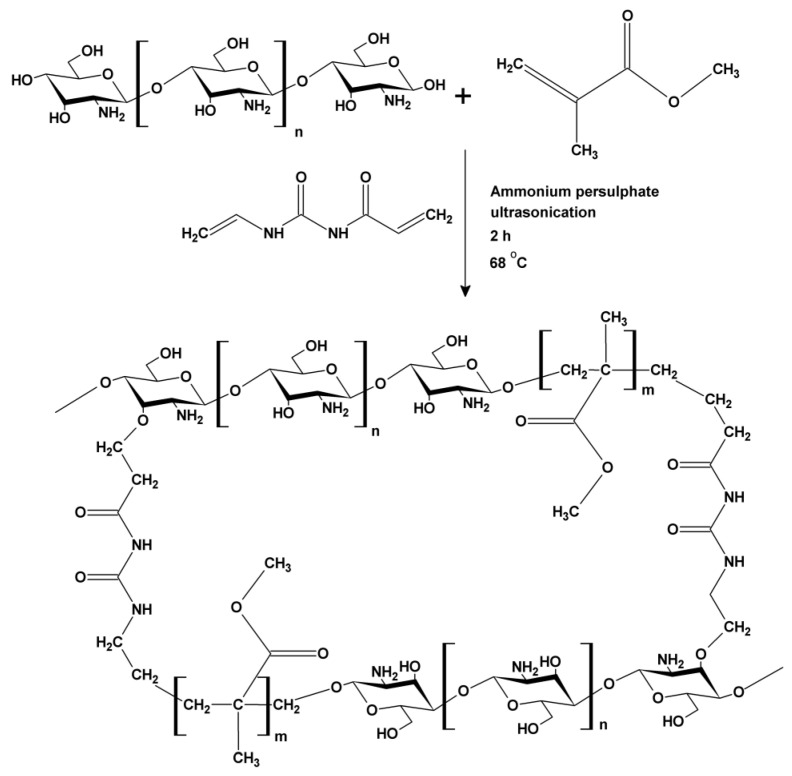
Synthesis of chitosan-*cl*-poly(MMA) hydrogel nanospheres.

The four different samples of chitosan-*cl*-poly(MMA) nanohydrogel nanospheres were prepared by employing the ultrasonication method. Scheme 1 depicts the synthesis of chitosan-*cl*-poly(MMA) hydrogel nanospheres. The concentration of MMA was varied while the concentration of chitosan was kept constant. It was observed that by using the ultrasonication technique for the fabrication of nanohydrogels at a particular molar ratio of chitosan:MMA, perfect nanospheres were obtained without any deformation in shape. The FTIR spectra in Figure 1a show prominent peaks at 3429 cm^−1^ for -OH stretching; 3004 cm^−1^ for =C–H bonds; 2945 cm^−1^ for NH_2_ stretching; 2850 cm^−1^ for C-H bonds; 1727 cm^−1^, 1248 cm^−1^, 1194 cm^−1^, and 1148 cm^−1^ for symmetric and asymmetric stretching of C=O and C-O [23]; 1657 cm^−1^ for the carbonyl peak; 1528 cm^−1^ for the N-H peak; 1482 cm^−1^ for C-C stretching; 1448 cm^−1^ for CN ring stretching vibration; 1388 cm^−1^ for a-methyl group vibrations; 984 cm^−1^ for the bending of C-H bonds; 844 cm^−1^ for CO stretching; 750 cm^−1^ for C-N stretching vibrations; and 620 cm^−1^ for the liberational vibrations of OH, thus ascertaining the formation of chitosan-*cl*-poly(MMA) nanohydrogel spheres [24,25,26,27,28]. Figure 1b displays the X-ray diffraction patterns of various samples of chitosan-*cl*-poly(MMA) nanohydrogel spheres. All the samples appear to be semicrystalline in nature, with prominent peaks at 2θ values of 13°, 30°, and 40°. These 2θ values differ from the values for pure chitosan and polymethylmethacrylate but lie close to the original values (for polymethyl methacrylate 15°, chitosan 10°), showing the formation of chitosan-*cl*-poly(MMA) hydrogel nanospheres [29,30]. 

On the other hand, the microstructure of chitosan-*cl*-poly(MMA) hydrogels was also studied by proton nuclear magnetic resonance (1H-NMR), and spectra were compared with the corresponding chitosan and MMA spectra, as displayed in Figure 1c. The spectrum of chitosan shows that the peak is located at 2.1 ppm, which can be attributed to the H-N bond, and subsequently, the –OH bond is observed at 1.8 ppm. Then, the structure of MMA is easily identified by the peak associated with the O-CH_3_ bond situated at 3.7 ppm, the C=C-CH_3_ bond located at 1.8 ppm, and, to a lesser extent, by the peak placed at 2.3 ppm related to the CH_3_ bond [29]. Finally, the structure of the copolymer is represented by the O-CH_3_ bond and the -(-COO)-CH_3_ group, situated at 3.58 and 1.89 ppm, respectively [31]. In addition, -CH_2_ and –CH_3_ peaks are identified between 1 and 1.5 ppm. It was previously reported that the amino group is weakened due to some reactions associated with the new structure [29]. Hence, NMR data confirm that MMA was grafted onto chitosan, as the new spectrum exhibits, confirming the FTIR results. 

The thermal stability of the chitosan-*cl*-poly(MMA) hydrogels was analyzed by TGA, and the results are shown in Figure 1d. The thermal degradation of all samples occurs around 350 °C, as shown by TGA curves, but the presence of MMA seems to disrupt the stability of the gels [29,32]. In this context, the thermal residue is higher for the gel where the MMA content is lower, such that 23% residue was achieved for C1PM4 in comparison with C1PM10, where the final residue was around 3%. Thus, the presence of MMA could alter the thermal stability of the gels, which could contribute to the degradation of the samples.

**Figure 2:**
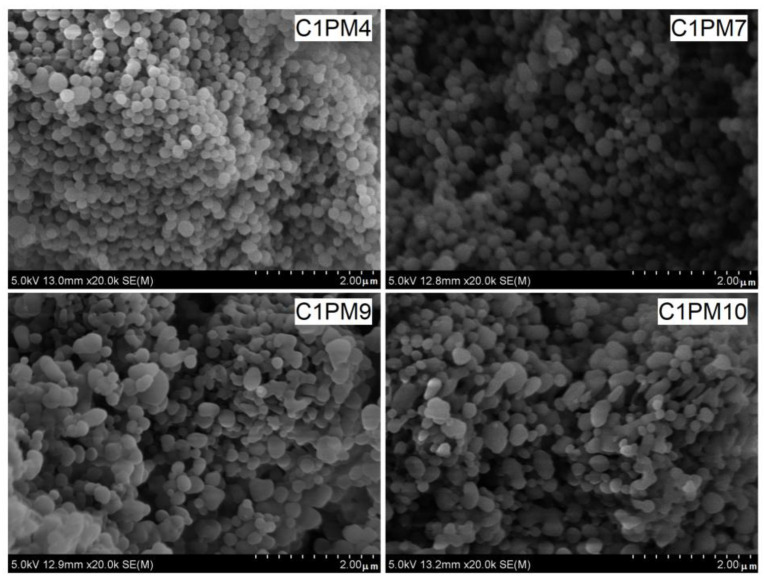
SEM micrographs of chitosan-*cl*-poly(MMA) hydrogels nanospheres.

**Figure 3:**
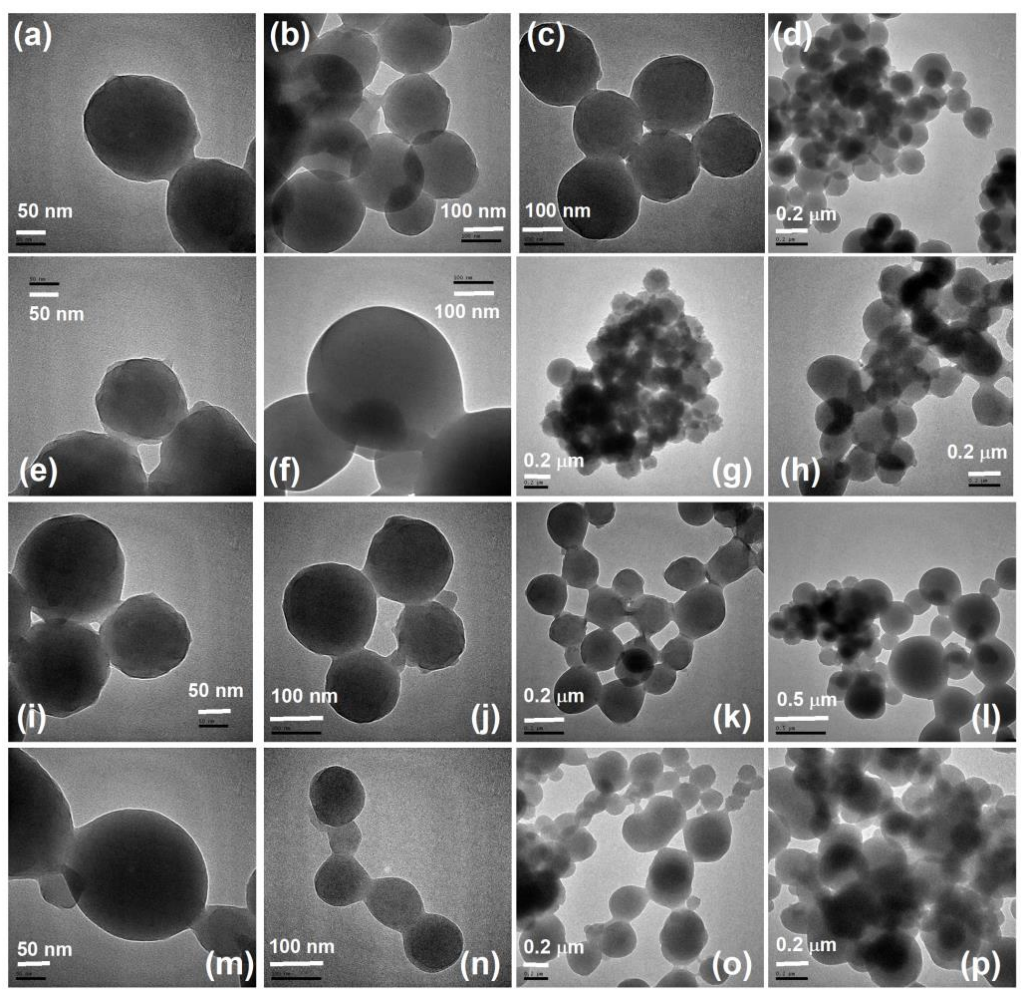
TEM images of (**a**–**d**) C1PM4, (**e**–**h**) C1PM7, (**i**–**l**) C1PM9, and (**m**–**p**) C1PM10 of chitosan-*cl*-poly(MMA) hydrogel nanospheres.

**Figure 4:**
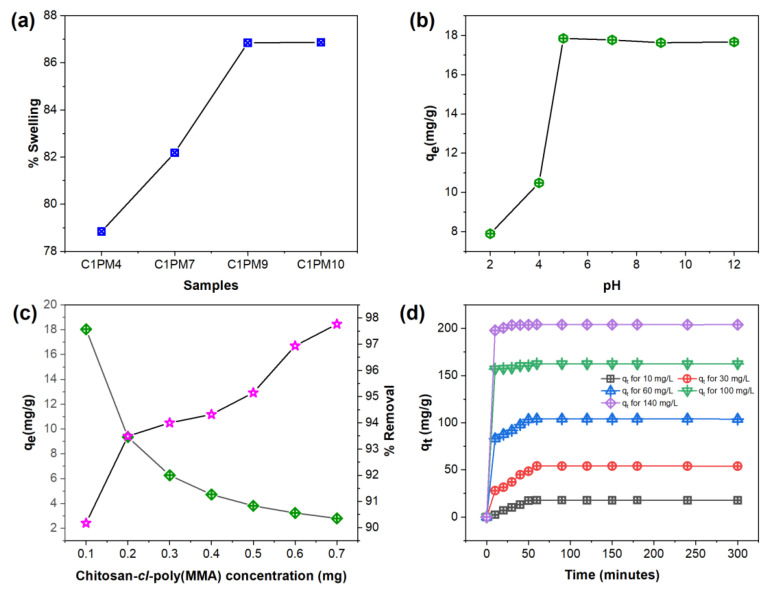
(**a**) Swelling percentage, (**b**) effect of pH, (**c**) effect of chitosan-*cl*-poly(MMA) nanohydrogel sphere concentration, and (**d**) effect of contact time and RhB dye concentration.

Figure 2 and Figure 3a–p depict the SEM micrographs and TEM images of all the samples of chitosan-*cl*-poly(MMA) hydrogels. It is clear from the images that all the samples possess a spherical shape in nano dimensions. The sample C1PM4 displays more regularly shaped nanospheres with a better smothering surface than those in the C1PM7, C1PM9, and C1PM10 samples. The swelling capacity of chitosan-*cl*-poly(MMA) hydrogel nanospheres is a vital characteristic that governs their probable uses in several applications [33]. The effect of dissimilar ratios of chitosan and MMA monomers on the swelling capacity of hydrogel nanospheres showed that the swelling percentage of synthesized C1PM10 hydrogel nanospheres with a ratio of 1:10 (after 24 h, the swelling percentage was 86.87%) was slightly better than that of other hydrogel nanospheres synthesized with ratios of 1:4, 1:7, and 1:9 (after 24 h, the swelling percentages of C1PM4, C1PM7, and C1PM9 were 78.83%, 82.18%, and 86.84%, respectively) (Figure 4a). The swelling properties of all the samples were comparable among themselves. For the adsorption sample, C1PM10 was further used. 

### 2.1. Adsorption of Rhodamine B onto Chitosan-cl-poly(MMA) Nanohydrogels Spheres

#### 2.1.1. Adsorption Parameters

##### Effect of pH

The pH of the solution is one of the most important factors in the adsorption of dyes from an aqueous medium. In the present study, the effect of pH on the removal of RhB dye from an aqueous medium was examined over the pH range 2–12, and the results are shown in Figure 4b. As is clear from Figure 4b, the adsorption capacity for RhB dye increased from 7.9 mg g^−1^ to 17.8 mg g^−1^ after increasing the pH from 2 to 5. Above pH 5, there was a slight decrease in the adsorption capacity. So, the optimum pH value was 5 for RhB dye adsorption using chitosan-*cl*-poly(MMA) nanohydrogel spheres. The adsorption of RhB onto chitosan-*cl*-poly(MMA) nanohydrogel spheres was attributed to the electrostatic attraction between the cationic RhB dye and the hydroxyl and carboxyl groups of chitosan-*cl*-poly(MMA) nanohydrogel spheres. Dye surface specification at different pH values, surface chemistries of the adsorbent, and competing adsorption mechanisms (attractive and repulsive binding) are only a few of the proposed causes of RhB’s apparent decrease in adsorption capacity above pH 5 [34,35]. The adsorption capacity is maximized at pH 5 due to electrostatic forces of attraction. The slight reduction in adsorption capacity may be due to these attractive forces becoming weaker above this pH [35].

##### Effect of Chitosan-*cl*-poly(MMA) Nanohydrogel Sphere Concentration 

The effect of chitosan-*cl*-poly(MMA) nanohydrogel sphere concentration on the removal of RhB dye (as shown in Figure 4c) was investigated over a concentration range of 0.1–0.7 mg. The graphical representation clearly illustrates a reverse correlation between adsorption capacity and adsorption percentage, illuminating the complex interplay driven by the concentration of chitosan-*cl*-poly(MMA) nanohydrogel spheres. The observed trend reveals a reduction in adsorption capacity from 18.03 mg/g to 2.8 mg/g, with a concurrent increase in adsorption percentage from 90.2% to 97.8% (Figure 4c). The number of active sites ought to have increased if more adsorbent had been added, according to the basic theory [36]. However, it has been found that too much adsorbent causes additional interparticle interactions, such as aggregation, which eventually reduces the surface that is available for adsorption and, as a result, causes saturation of the available adsorption sites [37]. Thus, the decrease in adsorption capacity with an increasing concentration of chitosan-*cl*-poly(MMA) nanohydrogel spheres is most likely due to decreased adsorption sites for RhB dye molecule interaction and binding because of nanosphere aggregation [38,39].

Here, we can conclude that lower concentrations provide freer adsorption sites, increasing adsorption capacity; higher concentrations limit adsorption sites, thus reducing adsorption capacity.

##### Effect of Contact Time and RhB Dye Concentration

The effect of dye concentration and contact time between RhB and chitosan-*cl*-poly(MMA) nanohydrogel spheres is an important factor for the assessment of the adsorption capability of nanohydrogel spheres in the adsorption process. The experiment comprised adjusting the initial quantity of RhB dye from 10 mg/L to 140 mg/L while measuring time from 0 to 300 min. The goal was to find the optimal conditions for RhB dye adsorption by chitosan-*cl*-poly(MMA) nanohydrogel spheres. The effect of time on the adsorption of rhodamine B dye using chitosan-*cl*-poly(MMA) nanohydrogel spheres is shown in Figure 4d. As shown in the figure, the adsorption capacity for rhodamine B dye increased sharply with the contact time, and the maximum adsorption capacity was noted within 60 min. The fast adsorption rate at the initial stage was due to the high driving force and rapid transfer of RhB dye molecules to the surface of chitosan-*cl*-poly(MMA) nanohydrogel spheres. The adsorption rate then gradually slowed down because the RhB molecules consumed all the active sites of the chitosan-*cl*-poly(MMA) nanohydrogel spheres. When the initial dye concentration was increased from 10 mg/L to 140 mg/L, the dye’s equilibrium adsorption capacity (at 60 min) increased significantly from 17.9 mg/g to 204.3 mg/g, as shown in Figure 4d [39,40]. This emphasizes the importance of the initial concentration in determining adsorption capacity. 

#### 2.1.2. Adsorption Kinetics and Isotherm Modeling

##### Adsorption Kinetics

The most crucial aspect of designing an adsorption system is likely predicting the rate at which adsorption occurs for a particular system, with the kinetics of the system controlling the adsorbent size and adsorbate residence time. Analysis of the kinetics data is crucial since it aids in the prediction of the adsorption process and rate-control procedures. 

In the current work, the pseudo-first-order, pseudo-second-order, and intra-particle diffusion kinetic models were used to analyze the kinetics of the adsorption process. The physisorption and chemisorption natures of the adsorption process are indicated by the pseudo-first-order kinetics and pseudo-second-order kinetics, respectively. According to intra-particle diffusion kinetic models, which take process surface diffusion and mass transfer into account, intra-particle diffusion is the rate-regulating stage of adsorption [41,42]. For this investigation, non-linear forms of the pseudo-first-order (3), pseudo-second-order (4), and intra-particle diffusion kinetic (5) models were utilized. Their equations [41,42,43,44] are shown as

(1) $$q_{t}=qe(1-e^{-K_{1}t})$$

(2) $$q_{t}=\frac{{q_{\begin{array}{c} e\; \end{array}}}^{2}K_{2}t}{{1+qeK}_{2}t}$$

(3) $$q_{t}=K_{d}\sqrt{t}+C$$

where K_1_, K_2_, and k_d_ represent the rate constants of pseudo-first-order, pseudo-second-order, and intra-particle diffusion kinetic models, respectively. qe and qt are the amounts of dye adsorbed per unit mass of adsorbent (mg/g) at equilibrium and at some time t, respectively. C is the constant that represents interlayer thickness.

**Figure 5:**
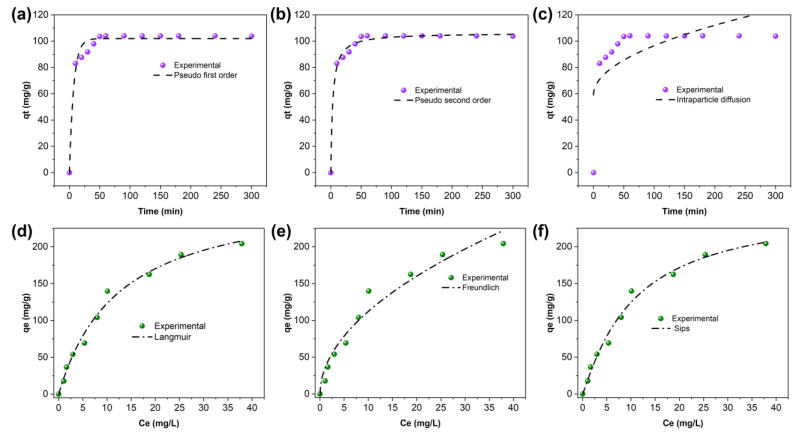
(**a**) Pseudo-first-order, (**b**) pseudo-second-order, (**c**) intra-particle diffusion, (**d**) Langmuir isotherm, (**e**) Freundlich isotherm, and (**f**) Sips isotherm for adsorption of RhB dye onto chitosan-*cl*-poly(MMA) nanohydrogel spheres.

It can be seen in Table 1 (Figure 5a–c) that the value of the correlation coefficient (R^2^) was better fitted for pseudo-second-order kinetics (0.9922) than for pseudo-first-order kinetic (0.9747) and the intra-particle diffusion kinetic (0.39973) models. The fact that the data is not well fitted for the intra-particle diffusion kinetic model indicates that intra-particle diffusion is not the rate-controlling step for the adsorption of the RhB dye by the chitosan-*cl*-poly(MMA) nanohydrogel spheres. The positive value of constant C (59.05) indicates the boundary layer effect of adsorption processes [45], and suggests that adsorption is controlled by some other mechanism apart from intra-particle diffusion [44]. The higher value of R^2^ indicates that the adsorption of RhB dye onto chitosan-*cl*-poly(MMA) nanohydrogel spheres follows pseudo-second-order kinetics. Also, the theoretical equilibrium adsorption capacity (qe = 106.597 mg/g) calculated from pseudo-second-order kinetics is in close proximity to the experimental data (qe = 104.087 mg/g). Based on these results, it was determined that the pseudo-second-order kinetics model best described the adsorption of the RhB dye by the chitosan-*cl*-poly(MMA) nanohydrogel spheres. A detailed investigation was carried out to understand the reaction kinetics results and is presented in Supplementary Materials, in the section 

**S1. Methods for validation of kinetic models in which we have used under the subsections**.

**S1.1. Non-linear kinetic model fitting:** The chi square value is less for pseudo second order model (6.2785) than pseudo first order model (20.292). Based on these results, it was determined that the pseudo-second order kinetics model best described the adsorption data.

**S1.2. Linear kinetic model fitting:** The linear kinetic model analysis, (Figure S1) depicts huge divergence in R^2^ and qe values, the data matches the pseudo-second-order kinetic model remarkably well, implying that this model provides a considerably better explanation of the underlying kinetics of the adsorption process details are given in Section S1.2.

**S1.3. Reduced non-linear equation fitting:** The PFO and PSO models can be reduced to equations using fractional uptake [46,47]. The considerable disparity in R^2^ values of PFO and PSO using reduced equations strongly suggests that the data matches exceptionally well with the pseudo-second-order kinetic model, implying that this model provides a considerably superior explanation for the kinetics details are given in Section S1.3.

**S1.4. Normal probability plots:** The data was analyzed by using normal probability plot (Figure S3). The construction of these plots was done according to calculations given in [46]. According to the normal probability plot, the pseudo first order has an unacceptable normal probability because the plot does not follow a straight line, whereas the pseudo second order, which does follow a straight line, is considered a valid model for studying the data details are given in section S1.4.

##### Adsorption Isotherm 

Adsorption isotherms describe the interactive behavior between the adsorbent (chitosan-*cl*-poly(MMA) nanohydrogel spheres) and adsorbate (RhB dye) molecules. To construct the adsorption system and assess the applicability of the adsorption process, adsorption isotherms, Langmuir, Freundlich, and Sips isotherms have been used to explain the equilibrium properties of adsorption [48]. The Langmuir and Freundlich models are two-parameter models, in which one explains monolayer adsorption (homogenous) and the other explains multilayer adsorption (heterogeneous), respectively. The non-linear equations of the Langmuir isotherm (6) and Freundlich isotherm (7) [48,49,50,51] are given as

(4) $$q_{\begin{array}{c} e\; \end{array}}=\frac{q_{max}K_{L}C_{e}}{1+(K_{L}C_{e})}$$

(5) $$q_{\begin{array}{c} e\; \end{array}}=K_{F}{C_{e}}^{\frac{1}{n}}$$

where Ce is the concentration of the dye solution at equilibrium (mg/L), and qe is the amount of dye adsorbed per unit mass of adsorbent (mg/g). K_L_ and K_F_ are a constant related to the free energy of adsorption (L/mg) and the Freundlich constant indicative of the relative adsorption capacity of the adsorbent (mg/g), respectively. q_max_ is the maximum adsorption capacity, and 1/n is the heterogeneity factor.

The Sips isotherm model is a three-parameter model with combined equations of the Langmuir and Freundlich models, and its non-linear Equation (6) [48,49,50,51,52] can be given as

(6) $$q_{\begin{array}{c} e\; \end{array}}=\frac{q_{s}K_{s}{C_{e}}^{n_{s}}}{1+(K_{s}{C_{e}}^{n_{s}})}$$

where K_s_ is the equilibrium constant. q_s_ is the Sips maximum monolayer adsorption capacity. n_s_ represents the Sips isotherm model exponent. This equation becomes a Langmuir equation if the value of n_s_ is equal to 1. Alternately, this isotherm lowers to the Freundlich isotherm as C_e_ or K_s_ get closer to zero.

The non-linear fitting curves and related fitting parameters of these isotherm models are shown in Figure 5d,e and Table 2. A comparison of these isotherm models shows that the adsorption data of RhB dye was better fitted by the Langmuir model, followed by the Sips model and the Freundlich model, due to the better correlation coefficient values of the Langmuir model (0.987), followed by those of the Sips (0.986) and then the Freundlich models (0.959). According to the Langmuir model of the adsorption isotherm, the adsorption reaches a saturation value beyond which no further adsorption takes place because of the monolayer, uniform, and finite adsorption site assumptions that underlie it. Additionally, it makes the assumption that molecules adsorbed by neighboring sites are not in communication with one another. Also, the value of the Sips isotherm model exponent n_s_ (1.089) is almost equal to unity, which means the Sips equation approaches the Langmuir adsorption isotherm, indicating monolayer adsorption of RhB dye by chitosan-*cl*-poly(MMA) nanohydrogel spheres. 

The maximum monolayer adsorption capacity (qmax) that was calculated using the Langmuir model was 276.26 mg/g, which is in close agreement with q_s_ calculated from the sips isotherm of 257.95 mg/g. The Langmuir model’s and Sips model’s predicted theoretical adsorption capacity values closely match the experimental values. In addition, to further determine whether the adsorption processes are favorable or unfavorable, the Langmuir dimensionless separation factor R_L_ [48] was determined by using Equation (7) given below.

(7) $$R_{\begin{array}{c} L\; \end{array}}=\frac{1}{1+(K_{L}C_{0})}$$

where K_L_ is the Langmuir constant of adsorption (L/mg) and C_0_ is the highest initial concentration of RhB dye (mg/L). The separation factor (R_L_), which has values between 0 and 1, is regarded as favorable for the adsorption process [48]. Our study’s separation factor (R_L_) values were below unity, indicating favorable adsorption of RhB dye onto chitosan-*cl*-poly(MMA) nanohydrogel spheres [49]. 

### 2.2. Adsorption Thermodynamics

**Figure 6:**
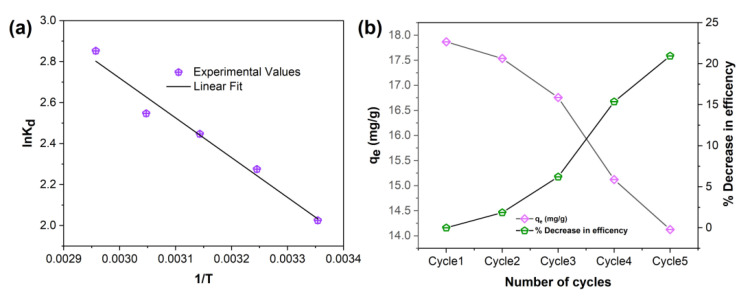
(**a**) The plot of lnK_d_ versus 1/T. (**b**) Reusability study for RhB dye adsorption onto chitosan-cl-poly(MMA) nanohydrogel spheres.

Thermodynamic factors deliver additional information concerning the inherent energy change and the mechanism of the adsorption process. The dimensionless standard equilibrium constant is required for the computation of ΔG°, ΔH°, and ΔS°. The standard state should transform the experimental equilibrium constant into the standard equilibrium constant [53]. For the adsorption of RhB dye using chitosan-*cl*-poly(MMA) nanohydrogel spheres, the values of various thermodynamic parameters like ΔG° (change in Gibbs free energy), ΔH° (change in enthalpy), and ΔS° (change in entropy) were determined at temperatures ranging from 25 °C to 65 °C by using the thermodynamic van ’t Hoff Equations (8) and (9) [53] given below.

(8) $$∆G{}^{\circ}=-RTlnK_{d}^{{}^{\circ}}$$

(9) $$lnK_{d}^{{}^{\circ}}=\frac{∆S{}^{\circ}\;}{R}-\frac{∆H{}^{\circ}}{RT}$$

where $K_{d}^{{}^{\circ}}$ is the standard equilibrium constant, R is gas constant (8.314 j/Kmol), and T (K) is the absolute temperature of the solution. ΔH° and ΔS° were determined by the slope and intercept of the van ’t Hoff plot of ln $K_{d}^{{}^{\circ}}$ vs 1/T, given in Figure 6a. 

According to Table 3, the reaction is exothermic, and the adsorption process is enthalpically favored based on the negative value of ΔH°. The enthalpy change caused by chemisorption is typically greater than that caused by physisorption and ranges from 40 to 120 kJ/mol [54]. As a result, this study’s low value for ΔH° (–16.13 Kj/mol) shows that physisorption is likely to be the cause of the adsorption and that the major type of contact between the RhB dye and the chitosan-*cl*-poly(MMA) nanohydrogel spheres is electrostatic (Columbic interactions). Only extremely weak intermolecular forces, such as van der Waals, and mostly electrostatic interactions are involved in the heat of physical adsorption.

The values of ΔG° at all temperatures were negative during the adsorption process, which confirmed that the adsorption of RhB dye onto chitosan-*cl*-poly(MMA) nanohydrogel spheres was spontaneous with favorable interactions between adsorbate and adsorbent. Additionally, the results revealed that the ΔG° values decreased as the temperature of the adsorption medium climbed, indicating that the adsorption process is more feasible at higher temperatures [55]. In addition, the positive values of ΔS° indicated decreased disorder at the solid–solution interface during the adsorption process of RhB dye onto chitosan-*cl*-poly(MMA) nanohydrogel spheres. The positive value of ΔS° indicates that RhB dye was not being adsorbed from a disordered state to an ordered one but rather at random at the solid–liquid interface [56]. This positive value also shows that structural modifications have occurred in the adsorbent material [55].

### 2.3. Reusability

In order to lower the overall cost of the adsorption process, an excellent adsorbent should also be highly reusable. Furthermore, due to cost constraints, the removal of adsorbed dye can be viewed as a significant method of reusing adsorbents. In this work, following RhB dye adsorption, the chitosan-*cl*-poly(MMA) nanohydrogel spheres were reused for another five consecutive cycles of adsorption, and the results are shown in Figure 6b. To evaluate the reusability of chitosan-*cl*-poly(MMA) nanohydrogel spheres, ethanol was used as the solvent. Before the reuse of the chitosan-*cl*-poly(MMA) nanohydrogel spheres, the adsorbed RhB dye was removed from the nanohydrogel spheres by thoroughly washing the chitosan-*cl*-poly(MMA) nanohydrogel spheres with an ethanol–water mixture.

After five cycles, the percent decrease in efficiency of adsorption of RhB dye onto chitosan-*cl*-poly(MMA) nanohydrogel spheres increased from 0% to 20.943%, while the adsorption capacity decreased from 17.9 to 14.1 mg/g. After each cycle, the adsorption capacity and percentage may decrease due to the occlusion of some active sites by dye molecules that are difficult to desorb due to interactions with the chitosan-*cl*-poly(MMA) nanohydrogel spheres. Hence, it can be concluded that chitosan-*cl*-poly(MMA) nanohydrogel spheres had substantial reusability, making them a cost-effective adsorbent for RhB dye removal from an aqueous medium.

### 2.4. Possible Mechanism of Adsorption

**Scheme 2:**
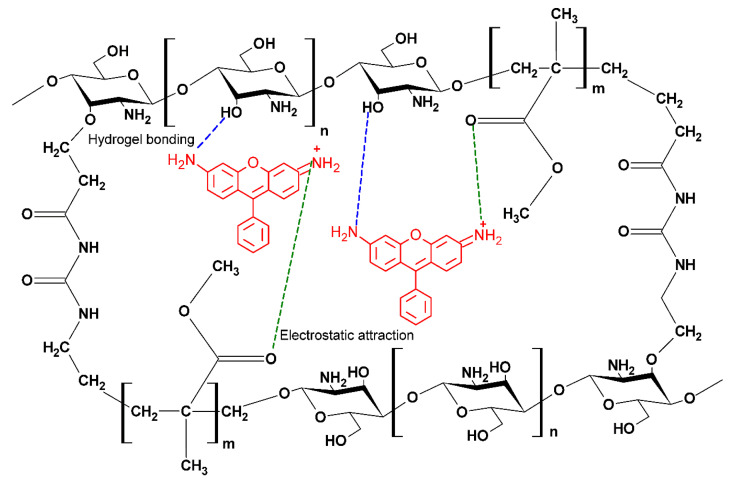
Possible interaction of RhB with chitosan-*cl*-poly(MMA) nanohydrogel spheres.

Based on the complete findings, the adsorption mechanism governing the interaction of RhB dye with chitosan-*cl*-poly(MMA) nanohydrogel spheres can be understood. According to the structural functional group, the significant adsorption capability highlights the presence of electrostatic interactions between the cationic RhB dye and the negatively charged hydroxyl and carboxyl ions on the surface of chitosan-*cl*-poly(MMA) nanohydrogel spheres [57]. The well-fitted pseudo-second-order kinetics and Langmuir adsorption isotherm indicate the critical importance of chemisorption sites on the hydrogel’s surface, indicating a chemisorption-driven mechanism [58]. This mechanism includes a variety of interactions, including electrostatic forces and covalent bonds. Furthermore, thermodynamic parameter analysis indicates the presence of physisorption behavior. This suggests that weaker interactions, such as van der Waals forces and hydrogen bonding, contribute to the adsorption process, including physical factors. In summary, the complex interaction between RhB dye and chitosan-*cl*-poly(MMA) nanohydrogel spheres involves a combination of physical and chemical interactions [58,59]. Electrostatic attractions caused by charge differences, hydrogen bonding assisted by functional groups, covalent bonding, and van der Waals forces are examples of these. This conglomeration of potential contacts is schematically shown in Scheme 2, highlighting the adsorption phenomenon’s intricacy.

## 3. Conclusions

In the present work, the usefulness of the ultrasonication technique for a shape-oriented hydrogel clearly reflects its potential. Thus, by using ultrasonication and varying monomer concentrations, we can design hydrogel spheres in nanodomains. The SEM and TEM analyses clearly depict the spherical geometry of all synthesized samples. The TGA analysis displays the higher thermal residue for the gel where the MMA content is lower, such as the 23% residue achieved for C1PM4 in comparison with C1PM10, where the final residue was around 3%. The swelling study indicates that higher MMA contents result in a higher swelling percentage. The adsorption potential of chitosan-*cl*-poly(MMA) nanohydrogel spheres was reflected by RhB dye adsorption onto them. The adsorption capacity for RhB dye utilizing chitosan-cl-poly(MMA) nanohydrogel spheres was pH dependent, increasing from 7.9 mg/g to 17.8 mg/g as pH climbed from 2 to 5, with a minor subsequent decline. The optimal pH for adsorption was found to be 5. Furthermore, altering the quantities of chitosan-*cl*-poly(MMA) nanohydrogel spheres resulted in a drop in adsorption capacity and an increase in adsorption percentage (90.2% to 97.8%). Adsorption capacity increased promptly with contact time, culminating within 60 min. Moreover, as the starting dye concentration increased, the equilibrium adsorption capacity increased. The adsorption results clearly display that chitosan-*cl*-poly(MMA) nanohydrogel spheres are novel adsorbents for dye removal, with a qmax value of 276.26 mg/g. The thermodynamics study reveals that chitosan-*cl*-poly(MMA) nanohydrogel spheres can be used as adsorbents over a broad range of temperatures. The isotherm, kinetics, and thermodynamics analyses reveal complex physio-chemical interactions involved in the adsorption process. Thus, it can be concluded that chitosan-*cl*-poly(MMA) nanohydrogel spheres are novel adsorbents that can be used for wastewater treatment.

## 4. Materials and Methods

### 4.1. Materials and Instruments

Chitosan, ammonium persulphate, methacrylate, N,N-methlenebisacrylamide, rhodamine B, and all other chemicals used were of analytical grade an obtained from Macklin. Double-distilled water was used for solution preparation.

### 4.2. Preparation of Various Samples of Chitosan-cl-poly(MMA) Hydrogel Nanospheres

The various samples of chitosan-*cl*-poly(MMA) hydrogel nanospheres were fabricated using ultrasonication technique. The chitosan:MMA ratios varied (details are presented in Table 4). Ammonium persulphate (5%) and N,N-methlenebisacrylamide (7%) were used as the initiator and crosslinker. The synthesis was carried out in an ultrasonic bath (Power:) for 2 h at 68 °C. To complete the polymerization, the samples were subjected to control stirring for 30 min at 70 °C. the obtained nanospheres were filtered and dried at 50 °C.

### 4.3. Characterization and Swelling Behavior

To investigate the chemical structure and morphology of chitosan-*cl*-poly(MMA) samples, different techniques were employed. The FTIR (Perkin Elmer Spectrum RX-IFTIR) spectra were obtained using the KBr method (400–4000 cm^−1^). The phase identification of chitosan-*cl*-poly(MMA) samples was performed using X-ray diffraction (XRD, SmartLab, Rigaku, Japan). The morphological studies were performed using scanning electron microscopy (SEM, SU-70, HITACHI, Tokyo, Japan) and transmission electron microscopy (F200, JEOL, Tokyo, Japan). To obtain the SEM micrographs, samples were coated with gold. For TEM analysis, a fine dispersion of chitosan-*cl*-poly(MMA) samples was dropped onto a carbon grid. The TEM micrographs were captured at an accelerating voltage of 200 KV. Thermogravimetric analysis was employed for measuring the weight loss of C chitosan-cl-poly(MMA) under the influence of temperature (or time) with a ramping rate of 10 °C/min. 

The samples of chitosan-*cl*-poly(MMA) were immersed in different flasks using tea bags (weight already measured), with each beaker containing 100 mL of distilled water at room temperature. The weight of the samples was noted after 24 h. The swelling percentage was calculated by the following equation:

(10) $$Swelling\;percentage=\frac{\left(S_{t}-S_{o}\right)}{S_{o}}\times 100$$

where $S_{o}$ is the weight of dried chitosan-*cl*-poly(MMA) samples and $S_{t}$ is the weight of chitosan-*cl*-poly(MMA) samples after time t. 

### 4.4. Adsorption of Rhodamine B onto Chitosan-cl-poly(MMA) Hydrogel Nanospheres

The adsorption of RhB dye onto chitosan-*cl*-poly(MMA) hydrogel nanospheres was investigated using the batch method. The influential adsorption parameters such as dye and chitosan-*cl*-poly(MMA) concentrations, contact time, pH, and temperature were first optimized. Finally, these optimized adsorption parameters were then employed for understanding the adsorption kinetics and also for calculating the maximum adsorption capacity of chitosan-*cl*-poly(MMA) hydrogel nanospheres. About 100 mL RhB dye solution was prepared in double-distilled water in a 100 mL Erlenmeyer flask. The flask was sealed and agitated at 200 rpm in a thermal incubator shaker at optimized values for time, pH, temperature, and other parameters. After adsorption accomplishment, the solution was filtered and centrifuged, and the concentration of RhB dye was confirmed by employing a double-beam UV-Vis spectrophotometer at 554 nm. The amount of rhodamine B adsorption per unit mass of chitosan-*cl*-poly(MMA) hydrogel nanospheres at time t and equilibrium were evaluated using the following balance equations [17,32]:

(11) $$q_{t}=(C_{0}-C_{t})\frac{V}{m}$$

(12) $$q_{e}=(C_{0}-C_{e})\frac{V}{m}$$

where, *qt* and *qe* are the amount of rhodamine B adsorbed at time t and equilibrium per unit weight of the chitosan-*cl*-poly(MMA) nanohydrogel spheres; *Co*, *C_t_*, and *C_e_* are the initial, at time t, and equilibrium concentrations of rhodamine B (mg/L); V is the total volume of solution (L); and m is total mass of chitosan-*cl*-poly(MMA) nanohydrogel spheres (g). 

Experiments for adsorption isotherms were performed with different concentrations of RhB dye solutions at 30 °C and 100 mg of chitosan-*cl*-poly(MMA) nanohydrogel spheres. Aliquots of these solutions are taken out at several time intervals, filtered, and analyzed by UV-Vis spectrophotometry. Similarly, kinetics experiments were performed.

## Figures and Tables

**Table 1:** Kinetic parameters for the adsorption of rhodamine B dye onto chitosan-cl-poly(MMA) nanohydrogel spheres.

**Pseudo-first-order**	**qe** **(mg g^−1^)**	**K_1_** **(min^−1^)**	**R^2^**
	101.98	0.14247	0.9747
**Pseudo-second-order**	**qe** **(mg g^−1^)**	**K_2_** **(g mg^−1^ min^−1^)**	**R^2^**
	106.59707	0.00294	0.9922
**Intra-particle diffusion**	**K_d_**	**C**	**R^2^**
	3.75	59.05	0.39973

**Table 2:** Isotherm model parameters for the adsorption of rhodamine B onto chitosan-cl-poly(MMA) nanohydrogel spheres.

Langmuir	Freundlich	Sips
Parameters	Parameters	Parameters
qmax (mg/g): 276.26	K_F_ (mg/g): 34.54	qs (mg/g): 257.95
K_L_ (L/mg): 0.08	n: 0.511	K_s_ (L/mg): 0.076
R^2^: 0.987	R^2^: 0.959	n_s_: 1.089
		R^2^: 0.986

**Table 3:** Thermodynamics parameters for the adsorption of rhodamine B.

ΔH° (KJ mol^−1^)	ΔS° (KJ mol^−1^ K^−1^)	ΔG° (KJ mol^−1^)				
		298.15K	308.15K	318.15 K	328.15 K	338.15 K
−16.13	71.00	−5.02	−5.83	−6.47	−6.95	−8.02

**Table 4:** Chitosan:MMA ratios for synthesis of chitosan-*cl*-poly(MMA) hydrogel nanospheres.

Sample Name	Chitosan (10%):MMA (Weight by Weight)
C1PM4	1:4
C1PM7	1:7
C1PM9	1:9
C1PM10	1:10

## Data Availability

Data is contained within the article or Supplementary Materials.

## Supplementary Materials

The following supporting information S1. Methods for validation of kinetic models results can be downloaded at: https://www.mdpi.com/article/10.3390/gels9100770/s1, Figure S1: linear curve fitting (a) pseudo first order kinetics, (b) pseudo second order kinetics., Figure S2: Non-linear curve fitting (a) reduced pseudo first order kinetics, (b) reduced pseudo second order kinetics. Figure S3: Normal probability plots (a) pseudo first order, (b) pseudo second order.
