# Supplementary material for: Tailoring Homogeneous Hydrogel Nanospheres by Facile Ultra-Sonication Assisted Cross-Linked Copolymerization for Rhodamine B Dye Adsorption
Source: Gels. 2023 Sep 22;9(10):770. doi: 10.3390/gels9100770 (PMC10606825; doi:10.3390/gels9100770)
Supplement: Supplementary file 1 [file gels-09-00770-s001.zip › gels-2574863-supplementary.pdf]

Supplementary information

Tailoring homogeneous hydrogel nanospheres by facile ultra-sonication assisted cross-linked copolymerization for rhodamine B dye adsorption

Gaurav Sharma [1*,2], Alberto García-Peñas [1*,3], Yaksha Verma [2], Amit Kumar [1,2], Pooja Dhiman [2] and Florian J. Stadler [1]

S1. Methods for validation of kinetic models results

S1.1. Non-linear kinetic model fitting:

We used nonlinear kinetic equations to study pseudo-first-order and pseudo-second-order kinetics in an examination of the data. The equations used are as follows:

$\mathbf{q_t = qe(1 - e^{-K_1 t})}$.................. (Non- linear pseudo first order equation)

$\mathbf{q_t = \frac{qe^{2} K_2 t}{1 + qe\, K_2 t}}$......................... (Non- linear pseudo second order equation)

the value of the correlation coefficient (R^2) was better fitted for pseudo-second order kinetics (0.9922) than pseudo-first order kinetics (0.9747). Also, the theoretical equilibrium adsorption capacity (qe = 106.597 mg/g) calculated from pseudo-second order kinetics is in close proximity to the experimental data (qe = 104.087 mg/g). Also, the chi square value is less for pseudo second order model (6.2785) than pseudo first order model (20.292). Based on these results, it was determined that the pseudo-second order kinetics model best described the adsorption data (figures 5a and b given in manuscript).

S1.2. Linear kinetic model fitting

The data was also analyzed by using linear kinetic equations for pseudo first order and pseudo second order kinetics. The equations used are following:

$\mathbf{\ln q_e - q_t = \ln q_e - K_1 t}$.............. (Linear pseudo first order equation)

$\frac{t}{q_e} = \frac{1}{q_e{}^2 K_2} + \frac{1}{q_e}$..................... (Linear pseudo second order equation)

Following linear regression analysis, we obtained a coefficient of determination (R^2) value of 0.999 for the pseudo-second-order response model. The R^2 value for the pseudo-first-order reaction model, on the other hand, was significantly lower at 0.203. Also, the theoretical equilibrium adsorption capacity (qe = 105.04mg/g) calculated from pseudo-second order kinetics is in close proximity to the experimental data (qe = 104.087 mg/g) (fig.S1). This huge divergence in R^2 and qe values suggested that the data matches the pseudo-second-order kinetic model remarkably well, implying that this model provides a considerably better explanation of the underlying kinetics of the adsorption process.

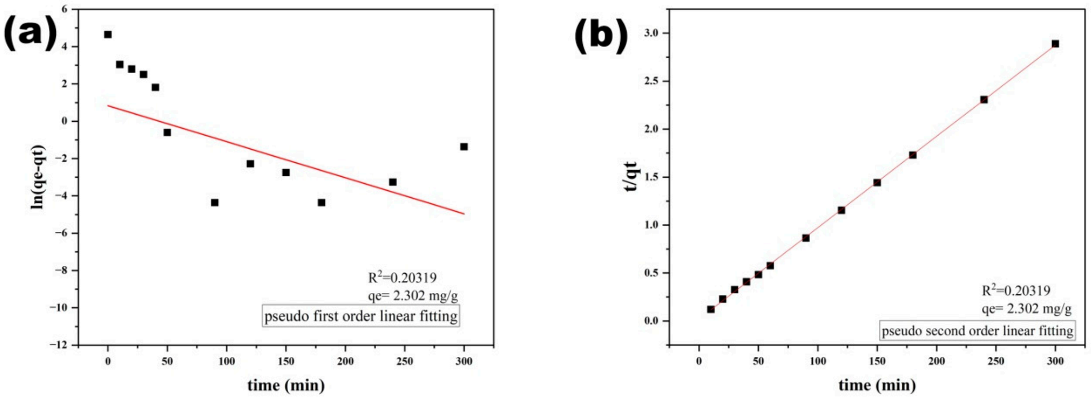

Figure S1. linear curve fitting (a) pseudo first order kinetics, (b) pseudo second order kinetics.

S1.3. Reduced non-linear equation fitting

A distinct approach to nonlinear modeling with PFO and PSO that used fractional uptake, F(t), defined as: $\mathbf{F(t)} = \frac{\mathbf{q(t)}}{\mathbf{q_e}}$

The PFO and PSO models can be reduced to equations using fractional uptake [1, 2].

$\mathbf{q(t)} = (\mathbf{1} - \mathbf{e}^{-\mathbf{K_1}\, t})$…………….. (Reduced pseudo first order equation).

$\mathbf{q_t} = \frac{{*\mathbf{K_2}}\, t}{\mathbf{1} + {*\mathbf{K_2}}\, t}$…………….. (Reduced pseudo second order equation), where $*\mathbf{K_2} = \boldsymbol{K_2 q_e}$.

After performing a nonlinear fitting analysis (fig.S2) and lowering the equation, we estimated a coefficient of determination (R^2) of 0.99023 for the pseudo-second-order response model. In comparison, the pseudo-first-order reaction model produced a significantly lower R^2 value of 0.97274. This considerable disparity in R^2 values strongly suggests that the data matches exceptionally well with the pseudo-second-order kinetic model, implying that this model provides a considerably superior explanation for the kinetics regulating the adsorption process.

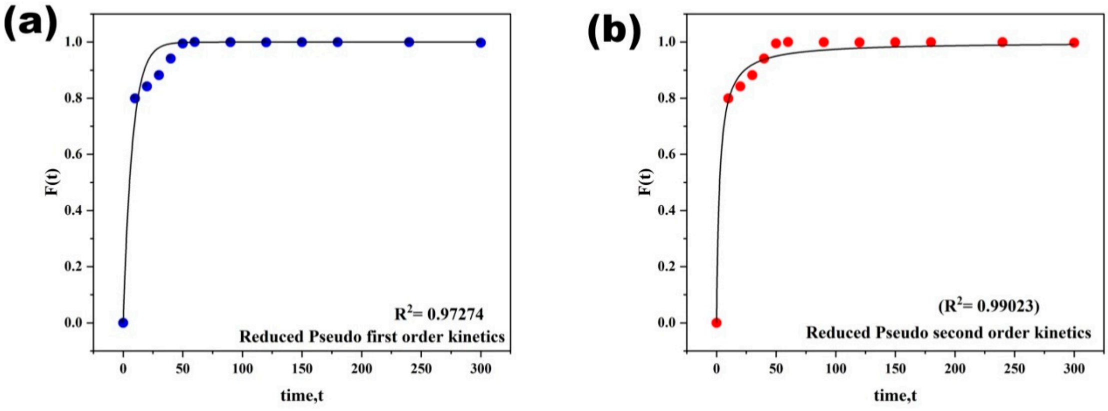

Figure S2. Non-linear curve fitting (a) reduced pseudo first order kinetics, (b) reduced pseudo second order kinetics.

S1.4. Normal probability plots

The data was analyzed by using normal probability plot (fig.S3); the residuals in a normal probability plot should approximate a straight line. When displaying the straight line, the center values of the plot should be prioritized over the extremes. Residuals are obtained by subtracting the observed values from the predicted ones from a statistical model. The construction of these plots was done according to calculations given in [1].

According to the normal probability plot, the pseudo first order has an unacceptable normal probability because the plot does not follow a straight line, whereas the pseudo second order, which does follow a straight line, is considered a valid model for studying

the data. Also, according to the literature, the residuals in a normal probability plot should approximately follow a straight line.

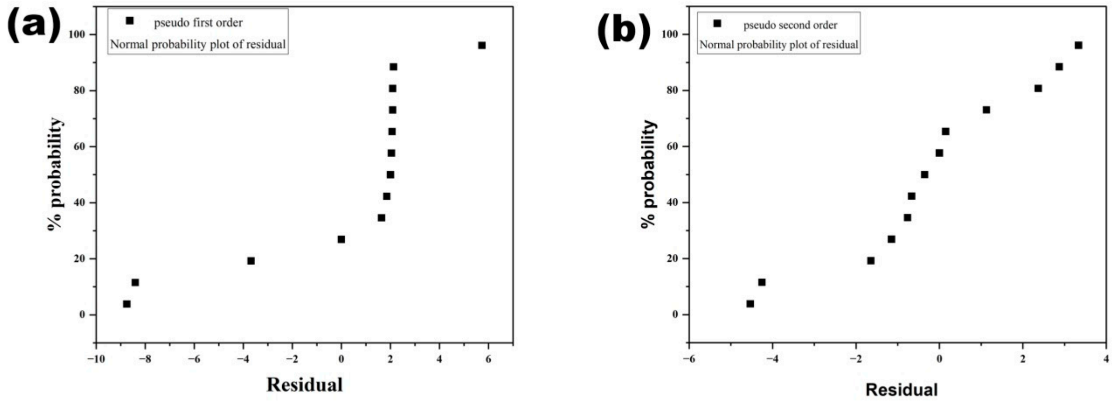

Figure S3. Normal probability plots (a) pseudo first order, (b) pseudo second order.

1. Revellame, E.D., et al., *Adsorption kinetic modeling using pseudo-first order and pseudo-second order rate laws: A review.* Cleaner Engineering and Technology, 2020. **1**: p. 100032.
2. Simonin, J.-P., *On the comparison of pseudo-first order and pseudo-second order rate laws in the modeling of adsorption kinetics.* Chemical Engineering Journal, 2016. **300**: p. 254-263.
